# Deep Learning-Based Automated Quantification of Coronary Artery Calcification for Contrast-Enhanced Coronary Computed Tomographic Angiography

**DOI:** 10.3390/jcdd10040143

**Published:** 2023-03-28

**Authors:** Jung Oh Lee, Eun-Ah Park, Daebeom Park, Whal Lee

**Affiliations:** 1 Department of Radiology, Seoul National University Hospital, Seoul 03080, Republic of Korea; 2 Department of Radiology, Seoul National University College of Medicine, Seoul 03080, Republic of Korea; 3 Department of Clinical Medical Sciences, Seoul National University College of Medicine, Seoul 03080, Republic of Korea

**Keywords:** coronary artery calcium score, coronary CT angiography, deep learning

## Abstract

Background: We evaluated the accuracy of a deep learning-based automated quantification algorithm for coronary artery calcium (CAC) based on enhanced ECG-gated coronary CT angiography (CCTA) with dedicated coronary calcium scoring CT (CSCT) as the reference. Methods: This retrospective study included 315 patients who underwent CSCT and CCTA on the same day, with 200 in the internal and 115 in the external validation sets. The calcium volume and Agatston scores were calculated using both the automated algorithm in CCTA and the conventional method in CSCT. The time required for computing calcium scores using the automated algorithm was also evaluated. Results: Our automated algorithm extracted CACs in less than five minutes on average with a failure rate of 1.3%. The volume and Agatston scores by the model showed high agreement with those from CSCT with concordance correlation coefficients of 0.90–0.97 for the internal and 0.76–0.94 for the external. The accuracy for classification was 92% with a 0.94 weighted kappa for the internal and 86% with a 0.91 weighted kappa for the external set. Conclusions: The deep learning-based and fully automated algorithm efficiently extracted CACs from CCTA and reliably assigned categorical classification for Agatston scores without additional radiation exposure.

## 1. Introduction

It is a well-established fact that the amount of coronary artery calcium (CAC) is a highly predictive indicator of cardiovascular events, both in the general population [1,2,3] and in diverse subgroups [4,5,6]. Accordingly, various methods have been attempted to quantify CAC: (a) a dedicated coronary calcium scoring CT (CSCT) as the reference [7]; (b) a dedicated CSCT using radiation reduction strategies, such as lower tube voltage, iterative reconstruction, or high-pitch scan acquisition [8,9,10]; (c) utilization of non-gated chest CT [11,12,13]; (d) virtual CSCT using dual-energy CT [14]; and (e) CAC extraction using enhanced ECG-gated coronary CT angiography (CCTA) [15,16,17,18,19,20,21,22,23,24]. Among them, CAC extraction using enhanced ECG-gated CCTA has the advantage of reducing radiation exposure by omitting the dedicated coronary calcium scoring CT. Several studies have demonstrated the usefulness of automated systems in quantifying CAC using contrast-enhanced CCTA [16,17,19,20,21,22]. However, those systems were associated with some limitations, including a high failure rate of 5–6% [17,25] and lack of diversity in tube voltages or CT machines [16,17]. We anticipated that our novel, fully automated, and deep learning-based approach, utilizing an adaptive threshold technique for calcium extraction and the weight-decision method for Agatston score computation, would effectively overcome these limitations and enhance the robustness of CCTA scans obtained at different kilovoltage peaks (kVps). Therefore, this study aimed to evaluate the accuracy of the deep learning-based automated quantification algorithm for CAC based on enhanced ECG-gated CCTA with a dedicated CSCT as the reference.

## 2. Materials and Methods

### 2.1. Data Collection

This retrospective study included a total of 315 individuals who underwent both CSCT and CCTA on the same day. The study population was divided into two groups: an internal validation group of 200 individuals who were examined at the Health Promotion Center and an external validation group of another 115 individuals who received the same examinations at Seoul National University Hospital. The Institutional Review Board of Seoul National University Hospital approved the study, and a waiver for the requirement of informed consent was granted. The individuals in the internal validation group were self-referred for a routine check-up from January to December 2019, and individuals in the external validation group were clinically scheduled for cardiac CT to evaluate coronary artery disease from October to December 2020. The exclusion criteria included age under 20 years, metallic stent insertion, and coronary bypass graft operation history. The automated algorithm for quantifying CAC was optimized using 202 additional CT scans from the same institution as the internal validation group and then tested on CT scans of the internal and external validation groups.

### 2.2. CT Image Acquisition

A second-generation dual-source CT scanner (Somatom Definition Flash; Siemens Healthineers, Forchheim, Germany) in Health Promotion Center and a first (Somatom Definition; Siemens Healthineers) or third-generation dual-source CT scanner (Somatom Force; Siemens Healthineers) in Seoul National University Hospital were used. Patients with a pre-scan heart rate of 70 beats per minute (bpm) or higher were given 100 mg of oral metoprolol (Betaloc; AstraZeneca, Cambridge, UK) 30–60 min before the CT examination unless the individual had a contraindication to beta-blockers. 

A CSCT scan was performed using prospective ECG-triggering with 70% of the R-R interval protocol (tube voltage, 120 kVp; tube current-time, 60 mAs; section thickness, 3 mm; increment, 3 mm; and filtered back projection). 

CCTA images were obtained after administration of 0.4 mg (patients ≥ 60 kg) or 0.2 mg (patients < 60 kg) of sublingual nitroglycerin (Nitroquick; Ethex, St. Louis, MO, USA) if not contraindicated. Sixty to eighty ml of a nonionic contrast medium (Xenetix 350; Guerbet, Aulnay-sous-Bois, France in the internal validation group and Iomeron 400; Bracco Diagnostics, Milan, Italy in the external validation group) was injected at a flow rate of 4–5 mL/s using a dual power injector (Stellant; Medrad, Indianola, IA, USA). The bolus tracking technique with a region of interest placed in the mid-ascending aorta was used to determine the timing of CT acquisition. The trigger threshold and delay for the prospective ECG-gated CT scans were 100 HU and 15 s, respectively; those for the high-pitch spiral scan (FLASH mode; Siemens Healthineers) were 150 HU and 8 s, respectively. 

The tube voltage and tube current were individually determined based on automated kVp selection software (CARE kV; Siemens Healthineers) and automatic exposure control (Care Dose 4D; Siemens Healthineers).

Parameters used for image reconstruction for CCTA included slice thickness of 0.75 mm, increment of 0.4 mm, and a kernel of I26f medium smoothness based on an iterative reconstruction algorithm (SAFIRE, strength 3, Siemens Healthineers) in the internal set. In the external set, slice thickness was 0.75 mm, the increment was 0.5 mm, and a medium smooth (Bv40) reconstruction kernel with an iterative reconstruction technique (ADMIRE strength 3, Siemens Healthineers) was used. Iterative reconstruction was not performed for CT scans of first-generation dual-source CT.

### 2.3. Reference Calcium Scores

The volume score [26] and Agatston score [7] were used to quantify CAC. Contiguous pixels of >1 mm^2^ with CT attenuation of >130 HU were quantified as calcifications. Briefly, the volume score represents the volume (mm^3^) of calcification, and the Agatston score is a weighted sum of the area of calcified plaques by peak attenuation of the plaques. The weights for the peak attenuations were as follows: 130–199 HU: 1; 200–299 HU: 2; 300–399 HU: 3; and ≥400 HU: 4. All CSCT images were transferred to a workstation (Syngo CT Workplace, Siemens Healthineers) and analyzed by an experienced radiologic technologist using dedicated software (Syngo Calcium Scoring, Siemens Healthineers) that is semi-automated and clinically established.

### 2.4. Automated Extraction and Quantification of Coronary Calcium on Contrast-Enhanced CCTA

Automatic segmentation and quantification of coronary calcium were performed using a dedicated cardiac prototype software (AutoSeg-H ver.1.1.005; AI Medic Inc., Seoul, Republic of Korea). The time required for computing calcium scores using the automated algorithm was recorded. 

**Figure 1:**
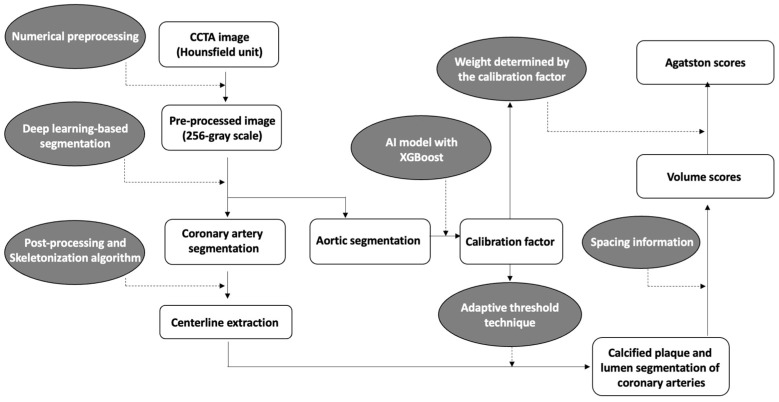
Overview diagram of the automated quantification method for coronary calcification.

**Figure 2:**
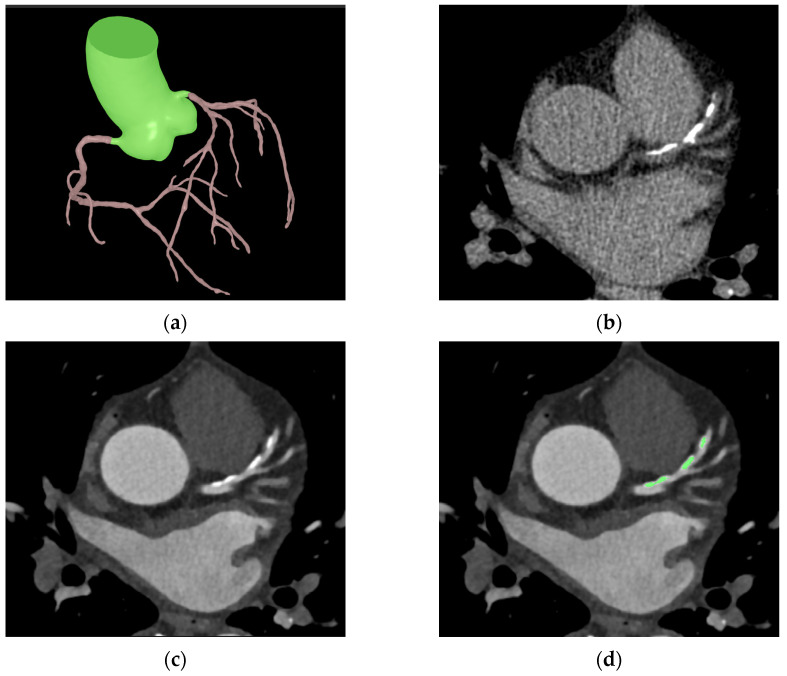
Sample images of automated coronary calcification segmentation. (**a**) shows a segmentation result of the coronary tree and ascending aorta; (**b**,**c**) show the same coronary calcification in CSCT and CCTA, respectively. The green area in (**d**) shows a segmentation result of the coronary calcification in CCTA.

A brief overview of the coronary calcium score acquisition in the software is illustrated in Figure 1. First, to enhance coronary vessels and facilitate segmentation, contrast-enhanced CCTA images were preprocessed by multiple numerical methods, including a Gaussian mixture model-based expectation-maximization algorithm [27,28], a newly developed attenuation histogram optimization algorithm, and Hessian filter application [29]. Using the preprocessed images, a two-dimensional deep learning network based on V-net [30] was trained in a 2.5-dimensional way to segment coronary arteries. Another neural network, Deeplab v3+ [31], was trained to segment the ascending aorta to obtain a calibration factor. The calibration factor was acquired from the attenuation values of the segmented ascending aorta via an artificial intelligence technique called XGBoost [32]. This calibration factor was not calculated as a simple mean attenuation of the green-highlighted aorta portion in Figure 2. Instead, the XGBoost technique was employed to extract a representative attenuation value of the coronary artery based on the attenuation values of the green-highlighted aorta portion used as inputs. 

Numerical post-processing methods were applied to coronary segmentation results to address missing or mislabeled voxels of the coronary trees. The location, distance, and type of coronary trees were used to connect the discontinuous vessels. Voxels mislabeled as coronary trees, such as veins or heart tissues, were eliminated by combining the angle, location, and attenuation information. The centerlines were extracted from the post-processed results using a skeletonization algorithm [33]. Then, using an adaptive threshold technique, which employs the previous calibration factor and pixel information for each cross-sectional plane of the centerlines, the software determined the boundaries of the coronary arteries’ lumen and calcified plaques (Figure 2). The calibration factor serves to compensate for potential inaccuracies in the coronary artery attenuation values obtained from cross-sections of the coronary artery centerline. For example, if calcium is present in the centerline, causing an increase in the attenuation value of the coronary artery, the threshold for calcium detection is determined using the calibration factor rather than the coronary artery attenuation value. In such cases, if the attenuation value exceeds 1.45 times the calibration factor, it is identified as calcium. On the other hand, if the cross-sectional coronary artery attenuation obtained through the centerline is accurately obtained, calcium is recognized if the attenuation of pixels is at least 1.25 times higher than the attenuation of the coronary artery centerline. 

From the previous segmentation results, a calcification volume for each coronary vessel was extracted using spacing information. For Agatston score calculation, an adaptive weight by attenuation of each calcification voxel was summed. The adaptive weights were determined by calibration factor because the factor reflects variability in attenuation of calcification by different tube voltages. The adaptive threshold technique and the weight-decision method were tuned using 202 additional cases with volume scores and Agatston scores obtained from CSCT as the reference. From segmentation of the coronary trees to quantification of coronary calcification, it took approximately five minutes per case on a personal computer equipped with an AMD Ryzen 5 5600X 6-Core Processor and a single NVIDIA GeForce RTX 3060 GPU.

### 2.5. Statistical Analysis

Continuous variables are presented as mean ± standard deviations, and categorical variables are described as frequencies with percentages. The continuous variables were compared using paired t-tests. Correlations were analyzed using Pearson correlation coefficients. The agreement between the CAC scores was evaluated using concordance correlation coefficients (CCCs) [34] and Bland-Altman plots. The categorical agreements of the Agatston scores were assessed using weighted kappa values [35,36]. Depending on the kappa and CCC values, the inter-method agreement was considered to be poor (0.20), fair (0.21–0.40), moderate (0.41–0.60), good (0.61–0.80), or excellent (0.81–1.00) [36]. The accuracy of the five categorical risk classifications for the automated quantification of CAC (auto-CAC) was analyzed. All statistical analyses were performed using R version 4.1.1, with the epiR v.2.0.36, vcd v.1.4-8, and blandr v.0.5.1 packages. A *p*-value of <0.05 was indicative of statistical significance.

## 3. Results

### 3.1. Dataset Characteristics

The baseline demographic characteristics of the internal validation group (*n* = 200) and external validation group (*n* = 115) are provided in detail in Table 1. In the internal validation set, the most common kVp used was 100, followed by 80 kVp, with 138 (69%) and 49 (24.5%) scans, respectively, while 13 (6.5%) scans were performed using 120 kVp. In the external validation set, the use of 100 kVp was also the most common with 85 (73.9%) scans, followed by 80 kVp with 24 (20.9%) scans, and 4 (3.5%) and 2 (1.7%) scans were performed using 120 kVp and 90 kVp, respectively.

### 3.2. Performance of Automated Quantification of Coronary Calcium on Contrast-Enhanced CCTA

The automated algorithm’s performance was assessed after excluding the failure cases from the analysis, with an overall failure rate of 1.3% (4 out of 315 cases), including two cases from the internal validation group and an additional two cases from the external validation group. The primary reasons for failure were respiratory motion artifacts and inappropriate pitch usage. The average time taken to compute CAC scores using the automated system was less than five minutes (292 s) with a standard deviation of 18.8 s.

**Figure 3:**
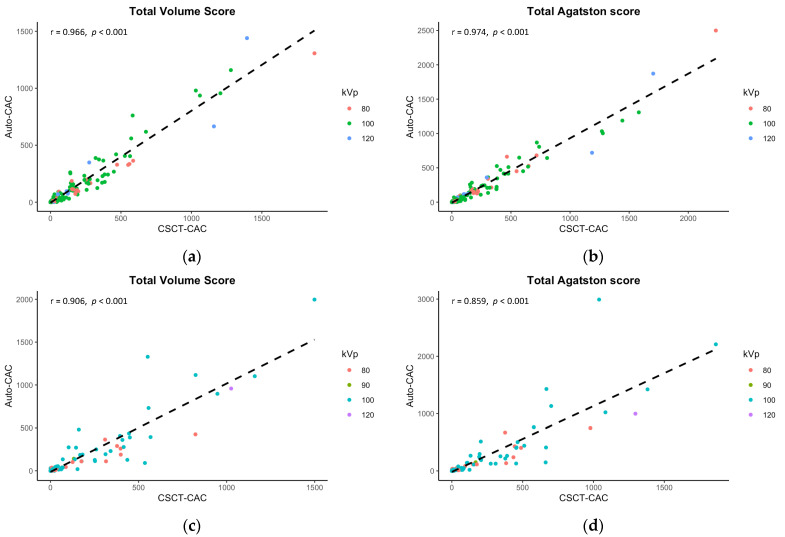
Correlations between the calcium scores in CSCT and the predicted calcium scores in CCTA. The internal validation results, presented in (**a**,**b**), demonstrate significant correlations in both the total volume score and Agatston score. Similarly, the external validation findings depicted in (**c**,**d**) reveal strong correlations in these same scores.

Table 2 summarizes the mean comparison and correlation/concordance coefficients of volume and Agatston scores acquired from dedicated CSCT and auto-CAC across anatomical locations (e.g., LCA, RCA, total) in the internal and external validation sets. Although there were significant differences in the mean scores between auto-CAC and CSCT in the internal validation set, no differences were found in the external validation set (*p* > 0.05 for both). In the internal set, the mean differences were −32.0 for the total volume scores and −17.8 for the total Agatston scores while in the external set, the mean differences were smaller at −8.4 and −9.2 for the volume and Agatston scores, respectively. The correlation between CSCT and auto-CAC was excellent for both the volume and Agatston scores, as shown in Figure 3. The concordance correlation coefficients were also excellent at any anatomical location in both the internal and external validation sets.

**Figure 4:**
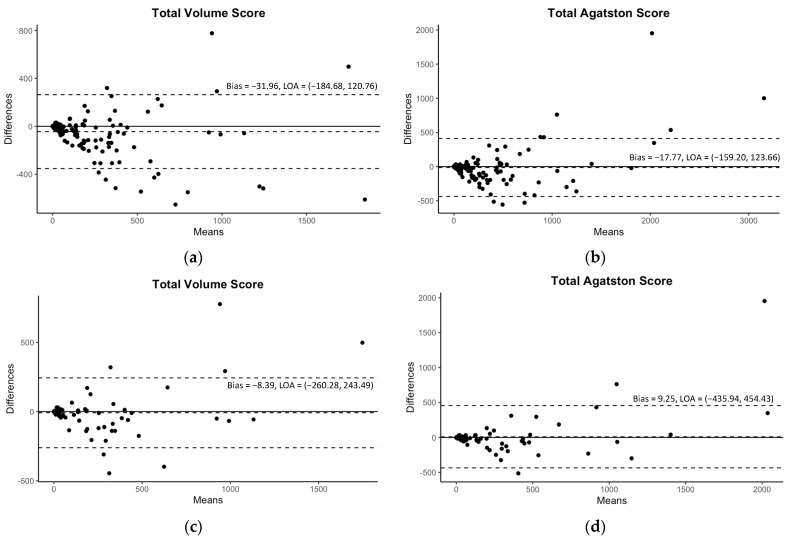
The Bland–Altman plots for the predicted volume scores and Agatston scores with 95% limits of agreement (LOA). The plots of the internal validation are presented in (**a**,**b**) for the total volume score and Agatston score, respectively. The plots of the external validation are presented in (**c**,**d**) for the same scores.

Although Table 3 demonstrated some significant differences in mean values of CAC scores between CSCT and auto-CAC, particularly in volume scores, there were still excellent correlations observed between CSCT and auto-CAC for both the volume and Agatston scores in both the internal and external validation sets as indicated in Table 3 and Figure 3. Similarly, the agreements were excellent at all kVp groups in both validation sets (Table 3). In the Bland-Altman plots in Figure 4, no specific pattern was observed in the difference between CSCT and auto-CAC, and most points were within 95% limits of agreement.

In Table 4, the categorical agreement of the Agatston scores was excellent in both internal and external validations. The classification accuracy was 91.9% with a 0.945 weighted kappa for the internal validation set. Among the participants, 16 (8.1%) were reclassified to a different cardiovascular risk category, with a majority of them (14/16, 87.5%) being shifted to a lower risk category. In the internal validation set, the accuracies by kVps were 90.5% and 95.8% for 100 kVp and 80 kVp, respectively. For the external validation set, the accuracy for classification was 85.8% with a weighted kappa of 0.906. Among the participants in the external validation set, 16 individuals (14.2%) were reclassified to a different cardiovascular risk category, and 9 out of 16 (56.2%) were shifted to a lower risk category. The accuracies by kVps were 88.2% and 78.3% for 100 kVp and 80 kVp, respectively, in the external validation set.

## 4. Discussion

The principal findings of this study are as follows: our deep learning-based automated model for quantifying CAC (a) extracted the volume and Agatston scores for the CAC efficiently from CCTA in five minutes with a failure rate of 1.3%, (b) demonstrated excellent correlation and agreement between estimated Agatston scores and the reference values, (c) constantly showed excellent performance regardless of kVp used, and (d) showed 86% accuracy for the five-risk classification in external validation.

Several studies have examined the use of contrast-enhanced CCTA for quantifying CAC and have reported a high correlation between the CAC scores extracted using automated models and the reference values, with a Pearson correlation ranging from 0.91 to 0.96 [15,16,17,18,19,20,21,22,23,24]. Among the studies that utilized fully automated CAC quantification models [16,17,19,20,22], only one was validated in external datasets [16,17], and our model showed a higher accuracy (86%) and a weighted kappa of 0.906 compared to previous studies, which reported accuracies ranging from 67% to 84% and weighted kappa values ranging from 0.784 to 0.906 for CAC risk classification. The high accuracy and adaptability of our model can be attributed to the application of the adaptive threshold method using the attenuation of the ascending aorta for identifying calcification, which enhances performance in preliminary studies [18,23]. Furthermore, our novel method had a lower failure rate (1.25%) compared to previous methods (5–6%) [17,25] in the segmentation step for coronary arteries, possibly due to the stability of deep learning-based segmentation compared to the previous numerical segmentation method.

Even though the amount of CAC obtained from CSCT is a well-known significant predictor of cardiovascular events in asymptomatic patients [1,2,3], its clinical significance has limitations in symptomatic patients [37,38,39,40]. Nevertheless, Hou et al. [41] found that combining CAC scores in CSCT and coronary stenosis degrees in CCTA provides a better prediction of cardiovascular events than using either alone in symptomatic outpatients, given the traditional risk factors. Considering that it is clinically common practice to perform two separate CT scans in patients with obstructive coronary artery disease (CAD) (CCTA to evaluate obstructive CAD and CSCT to quantify CAC), our automated algorithm can be of help to reduce radiation exposures. Reliable CAC score extraction from CCTA without a separate CSCT scan will effectively reduce radiation exposure in almost all patients with obstructive CAD; on average, the radiation would decrease by 0.5 mSv per patient if the CSCT scans were omitted from our dataset. Our fully automated algorithm will also reduce the workload of the radiographers for labor-intensive CAC score calculations. 

This study has several limitations. First, it should be noted that the absolute values of the extracted volume and Agatston scores obtained through the model were found to differ significantly from the reference values, which were generally smaller. This phenomenon is consistent with earlier studies that have also documented an underestimation of CAC on CCTA [16,19,24,41]. It is believed that the masking effect of the contrast agent on calcified plaques with similar attenuation may be the root cause of this underestimation. However, our model appears to have compensated for this effect when calculating Agatston scores, as evidenced by the smaller or even insignificant difference observed in these scores compared to volume scores. Furthermore, it is worth noting that our model demonstrated excellent performance in categorical classification, which is highly relevant from a clinical perspective when it comes to predicting cardiovascular events. Second, this was a single-center study, even though external validation test was conducted. Given that there can be variations in contrast and image acquisition protocols across institutions, it is possible that there could be variations in luminal attenuation as well, which could affect the accuracy of the model’s predictions. 

In conclusion, our deep learning-based and fully automated quantification algorithm efficiently extracted coronary calcium and reliably assigned categorical classification for Agatston scores from enhanced CCTA without additional radiation exposure.

## Figures and Tables

**Table 1:** Baseline characteristics of the internal and external validation sets.

Variables	Internal Validation Set (*n* = 200)	External Validation Set (*n* = 115)
Sex		
Women, *n* (%)	66 (33)	45 (39.1)
Men, *n* (%)	134 (67)	70 (60.9)
Age (years), mean ± SD	62.5 ± 8.57	62.4 ± 8.77
Body mass index (kg/m^2^), mean ± SD	24.5 ± 3.28	24.7 ± 3.06
Hypertension, *n* (%)	101 (50.5)	57 (49.6)
Diabetes, *n* (%)	64 (32)	28 (24.3)
Hyperlipidemia, *n* (%)	136 (68)	56 (48.7)
Family history of MI, *n* (%)	16 (8)	4 (3.5)
Smoking history		
Smokers, *n* (%)	32 (16)	17 (14.8)
Ex-smokers, > 1 month, *n* (%)	64 (32)	32 (27.8)
Non-smokers, *n* (%)	104 (52)	66 (57.4)
Heart rate (beat/min), mean ± SD	62.3 ± 9.37	65.2 ± 11.24

**Table 2:** Mean comparison and correlation coefficients between CAC scores by CSCT and Auto-CAC according to anatomical locations.

**Internal Validation**							
	**Comparison**			**Correlation**		**Agreement**	
**Parameters**	CSCT *	Auto-CAC *	***p* value**	***r* ^†^**	***p* value**	**CCC**	**95% CI**
Overall							
Volume score	142.12 ± 267.4	110.16 ± 224.2	<0.001	0.966	<0.001	0.942	0.928, 0.954
Agatston score	159.83 ± 316.8	142.06 ± 306.6	<0.001	0.974	<0.001	0.972	0.963, 0.978
LCA							
Volume score	102.22 ± 191.9	82.78 ± 176.6	<0.001	0.956	<0.001	0.947	0.931, 0.959
Agatston score	117.15 ± 230.4	105.40 ± 226.6	0.007	0.965	<0.001	0.963	0.952, 0.972
RCA							
Volume score	39.43 ± 112.8	27.38 ± 84.0	<0.001	0.952	<0.001	0.904	0.884, 0.921
Agatston score	43.14 ± 132.9	36.66 ± 137.6	0.007	0.970	<0.001	0.968	0.958, 0.976
**External validation**							
	**Comparison**			**Correlation**		**Agreement**	
**Parameters**	CSCT *	Auto-CAC *	***p* value**	***r* ^†^**	***p* value**	**CCC**	**95% CI**
Overall							
Volume score	150.63 ± 267.5	142.24 ± 303.7	0.48	0.906	<0.001	0.898	0.859, 0.928
Agatston score	178.22 ± 324.7	187.46 ± 433.9	0.66	0.859	<0.001	0.824	0.765, 0.869
LCA							
Volume score	98.76 ± 182.9	94.35 ± 196.6	0.46	0.947	<0.001	0.944	0.921, 0.961
Agatston score	119.6 ± 227.3	129.4 ± 342.7	0.60	0.826	<0.001	0.761	0.691, 0.817
RCA							
Volume score	51.87 ± 126.9	47.89 ± 156.7	0.62	0.835	<0.001	0.817	0.750, 0.867
Agatston score	58.62 ± 151.5	58.02 ± 185.6	0.94	0.896	<0.001	0.877	0.832, 0.911

CSCT, calcium scoring CT; auto-CAC, automated quantification of coronary artery calcium; CCC, concordance correlation coefficient; CI, confidence interval; LCA, left coronary artery; RCA, right coronary artery. * mean ± standard deviation; ^†^ Pearson correlation coefficient.

**Table 3:** Mean comparison and correlation coefficients between CAC scores by CSCT and Auto-CAC according to the tube voltages.

**Internal Validation**							
	**Comparison**			**Correlation**		**Agreement**	
**Parameters**	CSCT *	Auto-CAC *	***p* value**	***r* ^†^**	***p* value**	**CCC**	**95% CI**
80 kVp (*n* = 48)							
Volume score	141.80 ± 297.6	93.69 ± 203.7	0.001	0.993	<0.001	0.910	0.883, 0.930
Agatston score	161.50 ± 350.8	151.59 ± 383.7	0.27	0.990	<0.001	0.985	0.976, 0.991
100 kVp (*n* = 137)							
Volume score	131.78 ± 231.3	106.05 ± 205.2	<0.001	0.969	<0.001	0.956	0.941, 0.967
Agatston score	148.13 ± 277.8	127.56 ± 243.6	<0.001	0.977	<0.001	0.965	0.954, 0.974
120 kVp (*n* = 13)							
Volume score	252.25 ± 464.1	214.32 ± 415.1	0.35	0.955	<0.001	0.945	0.842, 0.981
Agatston score	276.99 ± 535.5	259.74 ± 526.0	0.67	0.964	<0.001	0.963	0.884, 0.988
**External validation**							
	**Comparison**			**Correlation**		**Agreement**	
**Parameters**	CSCT *	Auto-CAC *	***p* value**	***r* ^†^**	***p* value**	**CCC**	**95% CI**
80 kVp (*n* = 23)							
Volume score	135.29 ± 209.8	85.77 ± 129.7	0.009	0.929	<0.001	0.798	0.673, 0.878
Agatston score	157.77 ± 248.8	131.85 ± 219.9	0.05	0.908	<0.001	0.896	0.778, 0.952
100 kVp (*n* = 85)							
Volume score	149.73 ± 272.0	152.66 ± 330.3	0.08	0.915	<0.001	0.898	0.853, 0.929
Agatston score	176.84 ± 329.2	199.75 ± 478.0	0.17	0.866	<0.001	0.808	0.740, 0.860

CSCT, calcium scoring CT; auto-CAC, automated quantification of coronary artery calcium; CCC, concordance correlation coefficient; CI, confidence interval; LCA, left coronary artery; RCA, right coronary artery. * mean ± standard deviation; ^†^ Pearson correlation coefficient.

**Table 4:** Categorical agreement of Agatston score classifications by CSCT and auto-CAC.

**Internal validation**						
	**No. of patients in each risk classification by CAC Score from CSCT**					
		**0**	**1–10**	**11–100**	**101–400**	**>400**
No. of patients by auto-CAC	0	46	5	0	0	0
	1–10	0	15	6	0	0
	11–100	0	1	60	3	0
	101–400	0	0	0	39	0
	>400	0	0	0	1	22
**External validation**						
	**No. of patients in each risk classification by CAC score from CSCT**					
		**0**	**1–10**	**11–100**	**101–400**	**>400**
No. of patients by auto-CAC	0	27	4	1	0	0
	1–10	0	17	0	0	0
	11–100	0	5	21	1	0
	101–400	0	0	0	16	3
	>400	0	0	0	2	16

CSCT, calcium scoring CT; auto-CAC, automated quantification of coronary artery calcium.

## Data Availability

Not applicable.
